# The New Performance Calculation Method of Fouled Axial Flow Compressor

**DOI:** 10.1155/2014/906151

**Published:** 2014-08-13

**Authors:** Huadong Yang, Hong Xu

**Affiliations:** ^1^Department of Mechanical Engineering, North China Electric Power University, Baoding, Hebei 071003, China; ^2^School of Energy, Power and Mechanical Engineering, North China Electric Power University, Beijing 102206, China

## Abstract

Fouling is the most important performance degradation factor, so it is necessary to accurately predict the effect of fouling on engine performance. In the previous research, it is very difficult to accurately model the fouled axial flow compressor. This paper develops a new performance calculation method of fouled multistage axial flow compressor based on experiment result and operating data. For multistage compressor, the whole compressor is decomposed into two sections. The first section includes the first 50% stages which reflect the fouling level, and the second section includes the last 50% stages which are viewed as the clean stage because of less deposits. In this model, the performance of the first section is obtained by combining scaling law method and linear progression model with traditional stage stacking method; simultaneously ambient conditions and engine configurations are considered. On the other hand, the performance of the second section is calculated by averaged infinitesimal stage method which is based on Reynolds' law of similarity. Finally, the model is successfully applied to predict the 8-stage axial flow compressor and 16-stage LM2500-30 compressor. The change of thermodynamic parameters such as pressure ratio, efficiency with the operating time, and stage number is analyzed in detail.

## 1. Introduction

The performance of gas turbine is strongly influenced by environment conditions of the power plant. Gas turbine performance degradation over time is mainly due to the change of the blade profiles of compressor and turbine caused by fouling, corrosion, erosion, and foreign object damage (FOD). Among these degradation factors, compressor fouling is the most important reason for gas turbine performance deterioration. It is estimated that compressor fouling accounts for 70% to 85% of the gas turbine performance loss [1]. Even in the very clean conditions, compressor fouling has been confirmed to take place [2].

Particles, such as soil dust, pollen, seeds, and combustion products, mixed with oil vapors from internal and external leaks can readily adhere to the blade surface and annulus areas; thus, the shape of the airfoil is changed and the blade surface roughness is increased. The principle effect of compressor fouling is the reduction of mass flow rate, isentropic efficiency, pressure ratio, and power output.

Although fouling mechanism in axial flow compressor is well known, predicting to what extent the engine output and efficiency are affected is still a major challenge for performance engineers [3]. Linear progressive fouling model was developed by Aker and Saravanamuttoo [2] to investigate the effects of fouling on compressor and engine performance by modifying the appropriate stage flow and efficiency characteristics in a stepwise fashion. In MacIsaac's model [4], global severity factor and relative severity factors are developed for each individual stage in the compressor using a fault severity assignment table to model the compressor fouling. Rodriguez et al. [3] extended Aker's model based on adding a third coefficient accounting for an additional deterioration of the temperature rise coefficient.

Some authors offer criteria and mathematical models for fouling to characterize the compressor sensitivity to fouling. Tarabrin [5] suggested an index of axial compressor sensitivity to fouling. This approach allows evaluating the axial compressor sensitivity to fouling in order to accurately select appropriate washing schedule.

Melino et al. [6] developed a model to evaluate the performance degradation of an axial compressor due to fouling by using a stage-stacking method for the simulation of compressor behavior. The model is able to reproduce the change of axial compressor performance maps due to fouling through a scaling technique.

Mohammadi and Montazeri-Gh [7] simulated compressor fouling in the full and part-load conditions; from this paper it can be seen that fouling is not the same in different running load.

**Figure 1:**
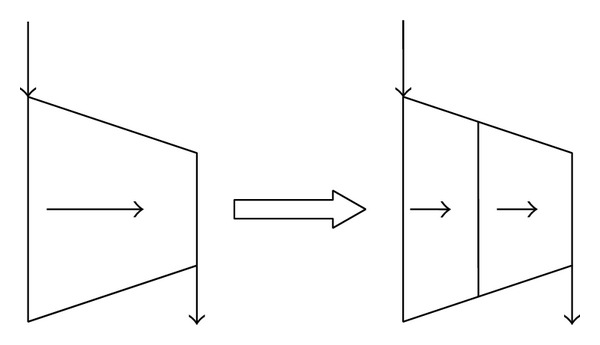
The diagram of sectionalized axial flow compressor.

The research revealed that fouling can progress into 40 to 50 percent of the compressor stages [2]. This paper presents a new performance prediction method to evaluate the effect of fouling on compressor performance during the steady operation. In this model, the compressor is divided into two sections which are shown in Figure 1. The first section includes the first 50% stages which represent the fouled stages and the remaining stages are considered as the second section which represent the clean stages. The performance of the fouled stages is predicted by combining scaling technique, linear progression model, and stage-stacking method; simultaneously this model can predict the performance of fouled compressor at specified operating time. However, the performance of the clean stages is estimated by averaged infinitesimal stage characteristics method.

## 2. Stage-Stacking Method

### 2.1. Stage Performance Parameters

The overall performance of clean multistage compressor can be evaluated which requires performance for every stage to be available. In order to simulate compressor stage performance, the following nondimensional parameters are used.

Flow coefficient is as follows:

(1) $$\begin{array}{c} \phi =\frac{C_{a}}{U}=\frac{V_{Z,1}/\sqrt{\theta }}{U_{m,1}/\sqrt{\theta }}. \end{array}$$

Pressure rise coefficient is as follows:

(2) $$\begin{array}{c} \psi =\frac{C_{P}T_{sl}\left({\text{PR}}_{S}^{(\gamma -1)/\gamma }-1\right)}{U^{2}},\\ U=\frac{U_{m,1}}{\sqrt{\theta }},\\ U_{m,1}=\omega r_{m,1}. \end{array}$$

Here, *C*_*a*_ is the stage inlet axial velocity, *U* is the tangential blade speed at mean radius, *V*_*Z*,1_ is the axial flow velocity in the inlet, *U*_*m*,1_ is the midspan rotor speed, *θ* is temperature ratio of inlet total temperature and standard sea-level temperature, *C*_*p*_ is specific heat at constant pressure, *T*_*sl*_ is the NASA standard sea-level temperature of 288.17 K, PR_*S*_ is the stage pressure ratio, *γ* is the ratio of specific heat, *ω* is the angular speed of rotating shaft, and *r*_*m*,1_ is the midspan radius at the stage inlet.

Temperature rise coefficient is as follows:

(3) $$\begin{array}{c} \sigma =\frac{C_{P}\Delta T_{OS}}{U^{2}}. \end{array}$$

Efficiency is as follows:

(4) $$\begin{array}{c} \eta =\frac{T_{OS}\left({\text{PR}}_{S}^{(\gamma -1)/\gamma }-1\right)}{\Delta T_{OS}}=\frac{\psi }{\sigma }. \end{array}$$

Here, *T*_*OS*_ is total temperature at the inlet and Δ*T*_*OS*_ is stage total temperature rise.

### 2.2. Stage Performance Curves

In order to model each compressor stage, generalized flow coefficient, generalized pressure rise coefficient, and generalized efficiency coefficient are used to evaluate the outlet performance parameters based on those parameters at inlet. Consider

(5) $$\begin{array}{c} \phi^{*}=\frac{\phi }{\phi_{\text{ref}}},\\ \psi^{*}=\frac{\psi }{\psi_{\text{ref}}},\\ \eta^{*}=\frac{\eta }{\eta_{\text{ref}}}. \end{array}$$

The selection of reference coefficient is very important to construct generalized stage characteristic curve. The reference coefficient (*ϕ*_ref_, *ψ*_ref_, *η*_ref_) can be obtained based on corresponding mass flow rate, pressure ratio, and temperature rise at maximum efficiency point [8].

**Figure 2:**
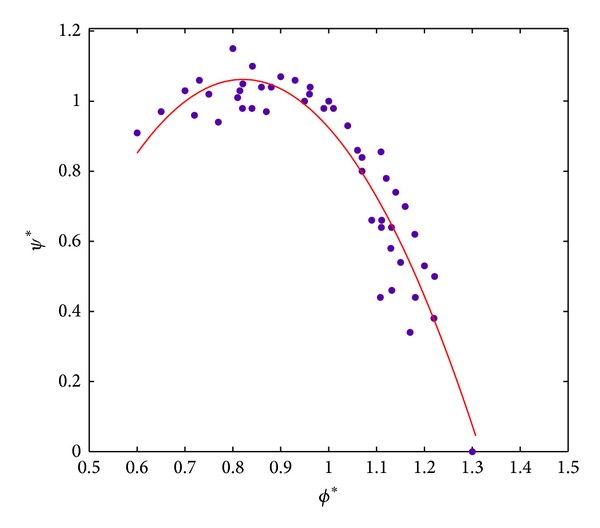
Generalized stage pressure coefficient curve.

Generalized pressure rise coefficient relationship was set up based on stage pressure rise data from a number of sources, which is shown in Figure 2 [8].

**Figure 3:**
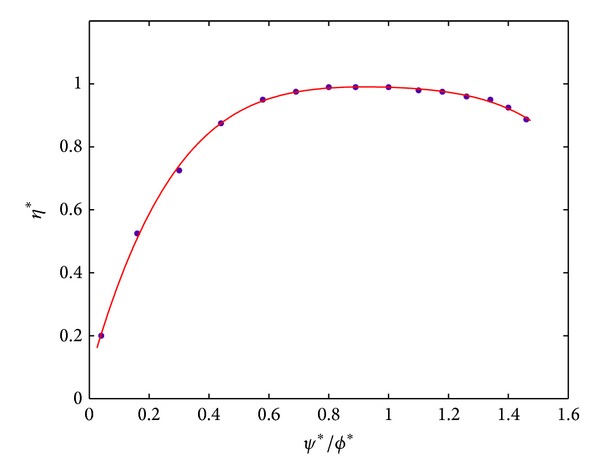
Generalized stage efficiency curve.

Generalized efficiency relationship is obtained from the curve proposed by Howell and Bonham [9], which is shown in Figure 3.

Once the generalized stage performance curve is available, the stage-stacking procedure is initiated by specifying compressor inlet parameters such as inlet flow coefficient, inlet total pressure, inlet total temperature, and compressor corrected speed. Thus, the outlet condition can be evaluated starting from the inlet ones.

## 3. Stage Performance Curve due to Compressor Fouling

Sandercock et al. [10–12] simulated the fouling of individual compressor stage by adjusting the stage flow and efficiency characteristics to reflect the effect of fouling on the engine performance. But the level of adjustment is arbitrary; many researches revealed that fouling is related to the operating time. This paper develops a model to characterize the relationship between the flow coefficient, pressure rise coefficient, efficiency, and operating time. Fouling can result in the reduction of mass flow rate, pressure, and efficiency, so that fouled compressor is operated at off-design condition. In order to predict the fouled compressor performance, the stage performance curve at off-design condition must be obtained.

The effect of fouling on stage performance parameters can be described using (6) based on the scaling techniques. The experiment data shows that the changes in mass flow rate and pressure ratio due to axial flow compressor fouling are nearly equal. The relation between the decrease in efficiency and the shift in pressure ratio due to fouling is described using (7). Consider

(6) $$\begin{array}{c} \phi_{f}=c_{\phi f}\cdot \phi_{\text{ref}},\\ \psi_{f}=c_{\psi f}\cdot \psi_{\text{ref}},\\ \eta_{f}=c_{\eta f}\cdot \eta_{\text{ref}}, \end{array}$$

(7) $$\begin{array}{c} c_{\phi f}=c_{\psi f},\\ c_{\eta f}=0.8c_{\psi f}. \end{array}$$

**Figure 4:**
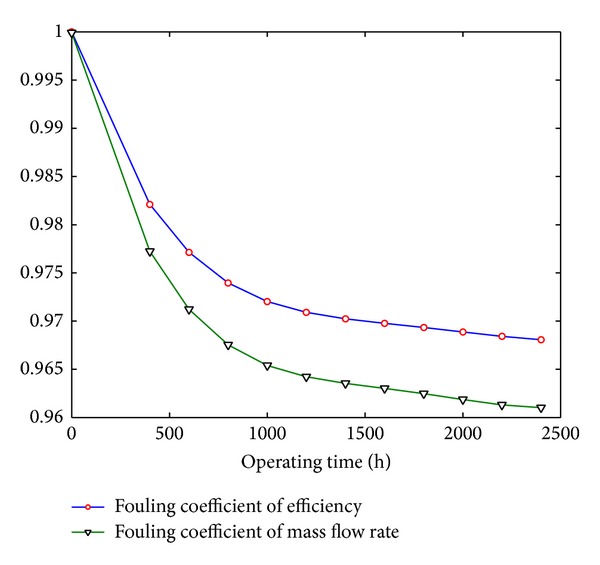
Fouling coefficients of mass flow rate and efficiency.

Here, *c*_*ϕf*_, *c*_*ψf*_, and *c*_*ηf*_ are fouling coefficients which depend on the operating time. The fouling level of compressor stage is represented by these coefficients. The lower these coefficients are, the more serious the fouling of compressor stage is. The fouling coefficient function is defined based on the literature [13] as shown in Figure 4. The literature data gives the trend between mass flow rate, pressure ratio, efficiency, and operating time (*f*_*ϕ*_(*t*), *f*_*ψ*_(*t*), *f*_*η*_(*t*)). Consider

(8) $$\begin{array}{c} c_{\phi f}=f_{\phi }\left(t\right),\\ c_{\psi f}=f_{\psi }\left(t\right),\\ c_{\eta f}=f_{\eta }\left(t\right). \end{array}$$

## 4. Stage Performance Degradation Model

At the same operating time, the performance parameters such as flow rate, pressure ratio, efficiency, and temperature rise are different for different stages. Because the deposited mass of particles is not uniform, the performance degradation of each stage is different. In order to accurately predict the effect of fouling on engine performance, fouling coefficient of each stage should be modified.

### 4.1. Fouling Severity Coefficient

Fouling level is affected by many factors such as particle concentration, particle size, particle material, temperature, and humidity. In this model, these influencing factors are considered by introducing fouling severity coefficient FS which is related to fouling coefficients *c*_*ϕf*_, *c*_*ψf*_, and *c*_*ηf*_.

Based on experiment data, the deposited particles are reduced from the first stage to the outlet, so the fouling severity coefficient for every stage should be different. In this model, linear progression model is applied based on the following assumptions, as shown in Table 1.

1. The fouling severity can be described by coefficient *r*_*s*_.
2. From the last research it can be seen that front stages of the compressors are more severely affected by fouling and the impact decreases linearly.
3. Only 40–50% of the compressor stages are affected.

So, the relative fouling severity coefficient for every stage FS_*i*_ can be determined by

(9) $$\begin{array}{c} {\text{FS}}_{i}=r_{si}\cdot {\text{FS}}_{g}. \end{array}$$

Here, FS_*g*_ is the global fouling severity coefficient taking values between 0 (new and clean) and 10 which is first determined based on ambient conditions.

Thus, stage performance parameters are modified as (10) using fouling severity assignment table, as reported in Table 2. Consider

(10) $$\begin{array}{c} \phi_{fi}=c_{\phi f}\cdot k_{\phi i}\left({\text{FS}}_{i}\right)\cdot \phi_{\text{ref}},\\ \psi_{fi}=c_{\psi f}\cdot k_{\psi i}\left({\text{FS}}_{i}\right)\cdot \psi_{\text{ref}},\\ \eta_{fi}=c_{\eta f}\cdot k_{\eta i}\left({\text{FS}}_{i}\right)\cdot \eta_{\text{ref}}. \end{array}$$

### 4.2. Configuration Coefficient

Cherkez [14] carried out the evaluation of the influence of the compressor fouling on the different schemes gas turbines with the same initial parameters. Research result shows that the two-shaft scheme and three-shaft scheme gas turbines are more sensitive to compressor fouling. It can be found that the relationship between stage performance parameters and operating time is different for different schemes. Therefore, configuration coefficient *c*_*g*_ is added to this model to increase the application range, as shown in Table 3.

Based on above analysis, the stage performance parameters are modified as follows:

(11) $$\begin{array}{c} \phi_{fi}=c_{\phi f}\cdot k_{\phi i}\left({\text{FS}}_{i}\right)\cdot c_{g}\cdot \phi_{\text{ref}},\\ \psi_{fi}=c_{\psi f}\cdot k_{\psi i}\left({\text{FS}}_{i}\right)\cdot c_{g}\cdot \psi_{\text{ref}},\\ \eta_{fi}=c_{\eta f}\cdot k_{\eta i}\left({\text{FS}}_{i}\right)\cdot c_{g}\cdot \eta_{\text{ref}}. \end{array}$$

## 5. Stage Performance of Clean Stages

Stages in downstream are recognized as clean stages, so the performance prediction can be performed based on averaged infinitesimal stage characteristics method. The second method decomposed compression process into limitless infinitesimal compression section based on the following assumptions.

1. The fluid state inside the compressor is characterized by a turbulent flow, which satisfies the self-modeling criterion. Subsequently, the Reynolds' dynamical similarity is satisfied automatically.
2. The change of density from the inlet to outlet of the stage is negligible, while the amount of infinitesimal stage tends to be infinite. Subsequently, similar work conditions satisfy both the geometrical similarity and kinematical similarity criteria.
3. The performance characteristics of each infinitesimal stage are similar; thus, different infinitesimal stages satisfy the same relative characteristic map.
4. Every stage denotes equal relative pressure and relative temperature increase ratio at the design point. Therefore, the efficiencies at the design point of each stage are equal.

In order to overcome the limitation of Reynolds' law of similarity, the compressor is imagined to be built up of a number of infinitesimal stages. Finally, the compressor's characteristic is calculated section by section. Based on Reynolds' law of similarity, the characteristics of each infinitesimal stage are being extrapolated, the performance curve of each infinitesimal stage is obtained, and furthermore the performance curve of the whole compressor is constructed. In this model, the second section is decomposed into 10 infinitesimal stages; the entrance parameter of the following stage is equal to the exit parameter of upstream stage [15].

The computation procedure is as follows.

1. The reference point of each stage is defined by design point.
2. The performance characteristics parameter at off-design condition is obtained based on Reynolds' law of similarity.
3. The stage performance is calculated based on above calculation result.
4. Finally, the total performance of axial flow compressor can be obtained.

### 5.1. The Averaged Stage Characteristics

Assuming that the compressor is decomposed into *z* infinitesimal stage, and the pressure ratio, temperature ratio of each infinitesimal stage is equal at design condition. And then the efficiency of each stage is equal to the efficiency of the whole compressor at design point. The total pressure and total temperature of the inlet of the second section are equal to the exit parameter of the first section. Consider

(12) $$\begin{array}{c} \pi_{i}={\pi_{2\text{nd}}}^{1/z}={\left(\frac{\pi }{\pi_{1\text{st}}}\right)}^{1/z},\\ \sigma_{i}={\sigma_{2\text{nd}}}^{1/z}={\left(\frac{\sigma }{\sigma_{1\text{st}}}\right)}^{1/z},\\ {\eta_{R}}_{i}=\frac{\gamma -1}{\gamma }\frac{\ln\pi_{2\text{nd}}}{\ln\sigma_{2\text{nd}}}. \end{array}$$

Here, *π* denotes pressure ratio, *σ* is temperature ratio, and *η* is efficiency. The subscript 1st denotes the first section and 2nd denotes the second section.

The flow coefficient of infinitesimal stage is

(13) $$\begin{array}{c} \phi_{R}=\frac{C_{R}}{U_{R}}. \end{array}$$

The stage characteristics at off-design condition can be obtained based on Reynolds' law of similarity by corrected mass flow rate and rotational speed. Consider

(14) $$\begin{array}{c} \pi_{i}={\left[1+\frac{f_{1}\left(\phi_{i}/\phi_{Ri}\right)}{f_{2}\left(\phi_{i}/\phi_{Ri}\right)}\cdot \left({\pi_{i}}^{\left(\gamma -1\right)/\gamma \eta_{i}}-1\right)\cdot {\left(\frac{N_{i}}{N_{Ri}}\right)}^{2}\right]}^{\gamma \eta_{i}/\left(\gamma -1\right)},\\ \eta_{i}=f_{2}\left(\frac{\phi_{i}}{\phi_{Ri}}\right)\cdot \eta_{Ri}. \end{array}$$

Here, *f*_1_  is compression work function, *f*_2_ is efficiency function

(15) $$\begin{array}{c} \frac{\phi_{i}}{\phi_{Ri}}=\frac{{\dot{m}}_{i}/{\dot{N}}_{i}}{m_{R}/N_{R}}. \end{array}$$

The second section performance is obtained by the following equation:

(16) $$\begin{array}{c} \pi_{2\text{nd}}=\overset{z}{\underset{i=1}{\prod }}\pi_{i},\\ \sigma_{2\text{nd}}=\overset{z}{\underset{i=1}{\prod }}\sigma_{i},\\ \eta_{2\text{nd}}=\frac{\gamma -1}{\gamma }\cdot \frac{\ln\pi_{2\text{nd}}}{\ln\sigma_{2\text{nd}}}. \end{array}$$

## 6. Performance Prediction Model of the Whole Compressor

The investigation of compressor blade contamination carried out at the Pervomayskaya gas piping compressor station [13] demonstrated that the deposited masses on blades are decreasing from the first stage to 50% stage. For the downstream stages, the deposited masses are nearly zero. Based on above experiment result, the actual fouled axial flow compressor is divided into two sections. The first section represents the fouled stages and the second section is viewed as clean stages.

Based on above discussion, after the performance was predicted, the whole compressor performance can be computed using

(17) $$\begin{array}{c} \pi =\pi_{1\text{st}}\cdot \pi_{2\text{nd}},\\ \sigma =\sigma_{1\text{st}}\cdot \sigma_{2\text{nd}},\\ \eta =\frac{\gamma -1}{\gamma }\cdot \frac{\ln\pi }{\ln\sigma }. \end{array}$$

## 7. Simulation Result Analysis

**Case 1 (8-stage axial compressor).**

In order to demonstrate the validity of stage-stacking method, an 8-stage axial compressor is applied. The detailed design parameters are as follows.

**Figure 5:**
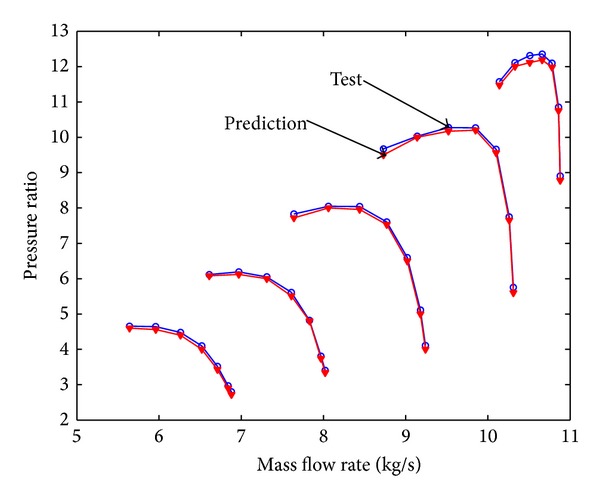
Pressure ratio characteristic curve for clean compressor.

**Figure 6:**
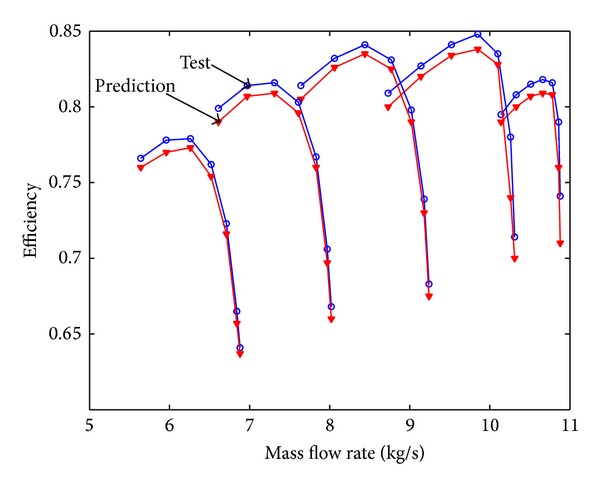
Efficiency characteristic curve for clean compressor.

Mass flow rate is 10.84 kg/s, air pressure at ambient conditions is 101.4 KPa and air temperature is 288 K. The total pressure ratio is 11.53 and polytropic efficiency is 88.8%. The clean compressor map is shown in Figures 5 and 6. From these figures it can be seen that the prediction curve based on stage-stacking method coincides well with design curve.

**Figure 7:**
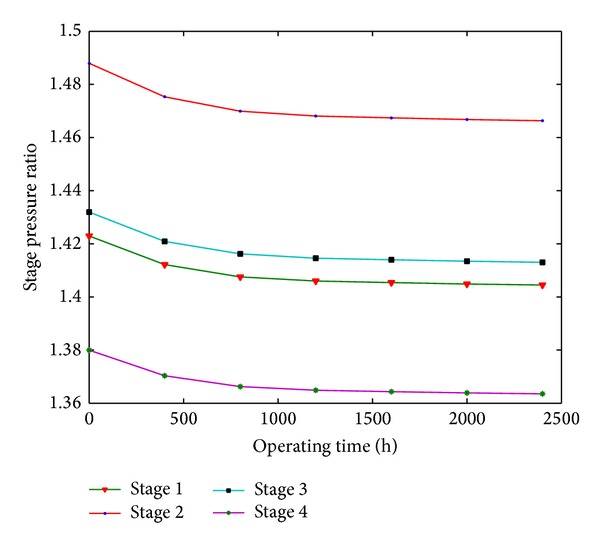
The relation between stage pressure ratio and operating time for fouled stages.

Based on fouling coefficient reference and fouling coefficient of each stage, fouled compressor performance can be calculated by modified stage-stacking method. The relation between stage pressure ratio and operating time is shown in Figure 7. From these figures it can be seen that stage pressure ratio is gradually reduced with the operating time. For stage 1, stage pressure ratio is reduced by 1.08% up to 800 hours and is reduced by 1.27% up to 2000 hours. For stage 2, stage pressure ratio is reduced by 1.21% up to 800 hours and is reduced by 1.42% up to 2000 hours. For stage 3, stage pressure ratio is reduced by 1.1% up to 800 hours and is reduced by 1.29% up to 2000 hours. For stage 4, stage pressure ratio is reduced by 0.99% up to 800 hours and is reduced by 1.17% up to 2000 hours.

**Figure 8:**
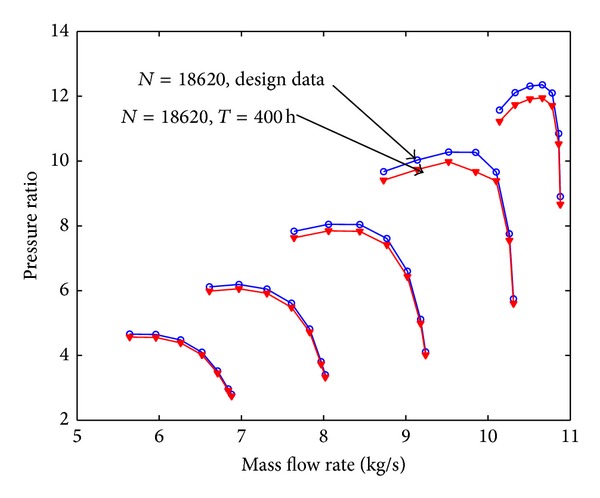
The pressure ratio curve in design and fouled condition when operating time is 400 hours.

**Figure 9:**
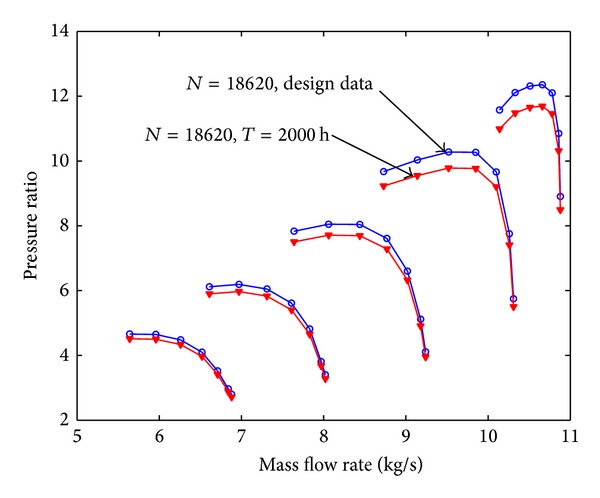
The pressure ratio curve in design and fouled condition when operating time is 2000 hours.

**Figure 10:**
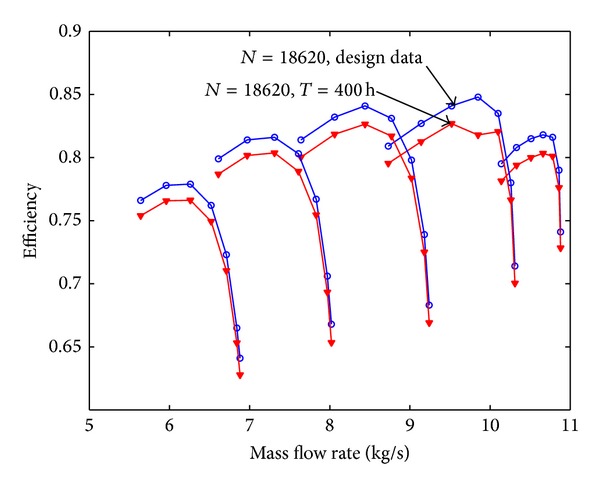
The efficiency curve in design and fouled condition when operating time is 400 hours.

**Figure 11:**
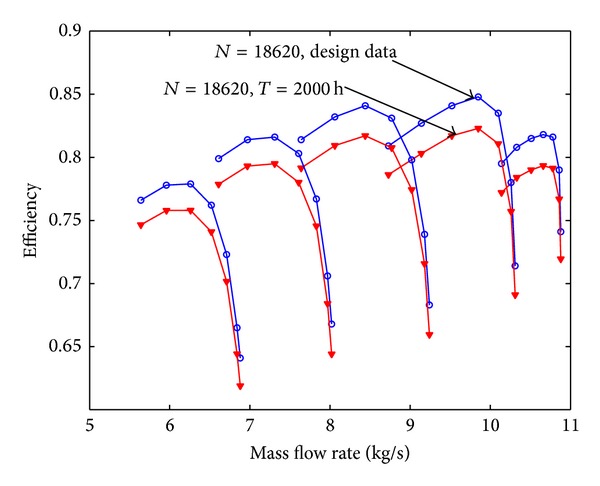
The efficiency curve in design and fouled condition when operating time is 2000 hours.

Assuming that the fouling level of the first four stages is uniform, the pressure ratio curve and the efficiency curve are shown from Figure 8 to Figure 11. Figures 8 and 9 show the relation between pressure ratio and mass flow rate in design and fouled condition when operating time is 400 hours and 2000 hours, respectively. From these figures it can be found that pressure ratio is different for different rotational speed. And the pressure ratio in design speed is mainly affected; the lower the speed is, the smaller the effect of pressure ratio is. Also, the pressure ratio is reduced with the increase of operating time. Figures 10 and 11 show the function of efficiency with mass flow rate for clean compressor and fouled compressor when operating time is 400 hours and 2000 hours. It is demonstrated that the efficiency is reduced with the increase of operating time. The variation of efficiency and pressure ratio is different. The efficiency is apparently reduced at any rotational speed.

**Figure 12:**
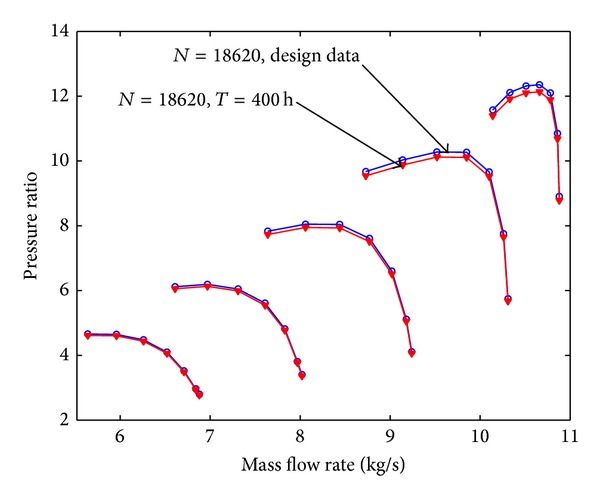
The pressure ratio curve with 400 hours due to fouling with different relative fouling severity coefficient.

**Figure 13:**
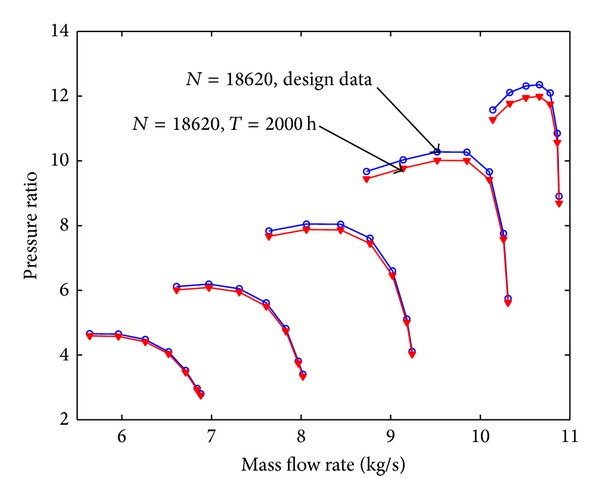
The pressure ratio curve with 2000 hours due to fouling with different relative fouling severity coefficient.

**Figure 14:**
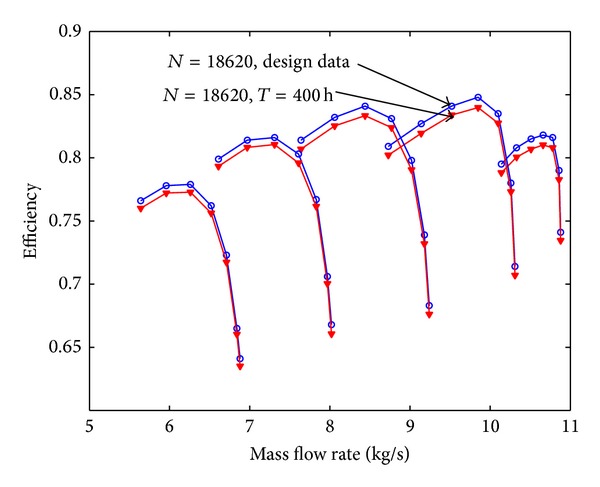
The efficiency curve with 400 hours due to fouling with different relative fouling severity coefficient.

**Figure 15:**
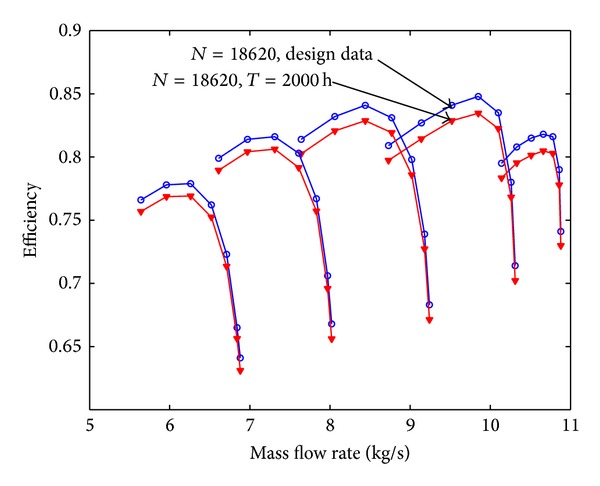
The efficiency curve with 400 hours due to fouling with different relative fouling severity coefficient.

The performance of fouled multistage axial compressor is computed based on the prediction model developed in this paper; the pressure ratio curve and efficiency curve are shown from Figures 12, 13, 14, and 15. On contrast to Figure 8, Figure 12 shows that the reduction value of these performance parameters is smaller with developed fouling model than those with uniform fouling. It coincides with the experiment data published in the literature and running data of real power unit.

**Case 2 (LM2500 compressor).**

**Figure 16:**
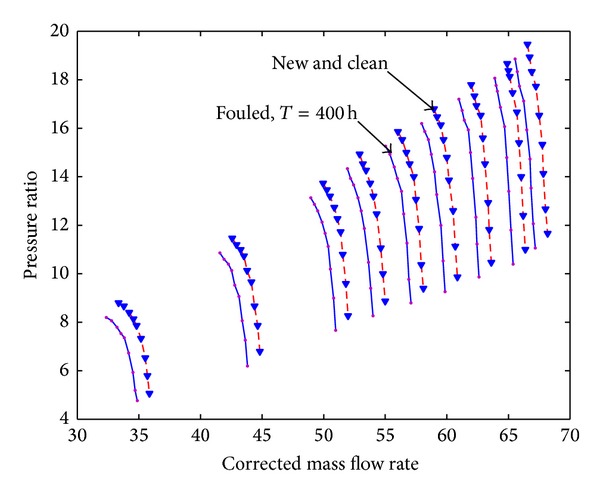
The pressure ratio curve of LM2500-30 with 400 hours due to fouling with different relative fouling severity coefficient.

The reference engine in this work is General Electric 2500 [10–12] aeroderivative gas turbine which is a tow-shaft engine with a sixteen-stage axial compressor (IGV and stators of stages one to six are variable geometry) and a free power turbine delivering 20 MW at 60 Hz.  Table 4 shows the design parameters of this engine. The operating line data of LM2500-30 is listed in Table 5. The model developed in this paper is applied to predict the performance of fouled LM2500-30 when it has been run for 400 hours. Compared with results shown in Figure 16 with results researched by many authors [3] can be seen that it is valid.

## 8. Conclusions

Fouling is the important influence factor of axial flow compressor performance degradation, so the prediction of the effect of fouling on compressor is crucial. In the previous research, CFD method and experiment study method are extensively used. Due to complex geometry and operating condition, it is very difficult to accurately predict the fouling phenomena.

This paper developed and validated a model which is able to evaluate the performance of fouled axial flow compressor. In multiple stage axial flow compressor, fouling is more serious in the first 50% stages than rear stages where particles deposited are less. So, the whole compressor is divided into two parts in this model. The performance of the first part is calculated by using stage-stacking method for the simulation of compressor behavior. The model is able to reproduce the change of axial compressor performance maps due to fouling through a scaling technique and linear progression model. Moreover, the introduction of the fouling sensitivity factor allows taking into account the different sensitivity of different compressors to the fouling.

The performance of second part is calculated based on averaged infinitesimal stage method. In view of the shortcomings of the conventional way of extrapolating performance curve of axial compressors, the concept of average stage cascade performance, together with some rational assumptions, is being presented. The compressor is imagined to be built up of a number of infinitesimal stages and the compressor's characteristic is calculated section by section.

The method was applied and tested using available data. The results of the application highlight the capability of the method to accurately predict the performance of multistage axial flow compressor due to fouling.

## Figures and Tables

**Table 1:** Linear progression model [5].

Step ↓	Stage 1	Stage 2	Stage *n*
	*r*_*s*1_	*r*_*s*2_	*r*_*sn*_
1	*k*_1_	—	—
2	2 · *k*_1_	*k*_1_	—
⋮	⋮	⋮	—
*n*	*n* · *k*_1_	(*n* − 1) · *k*_1_	*k*_1_

**Table 2:** Fouling severity assignment table [4].

FS	*k*_ϕ*i*_	*k*_ψ*i*_	*k*_η*i*_
0	1.00	1.00	1.00
1	0.96	0.99	0.95
2	0.92	0.98	0.90
3	0.88	0.97	0.85
4	0.84	0.96	0.80
5	0.80	0.95	0.75
6	0.76	0.94	0.70
7	0.72	0.93	0.65
8	0.68	0.92	0.60
9	0.64	0.91	0.55
10	0.60	0.90	0.50

**Table 3:** Configuration coefficient.

Engine configuration	*c*_*g*_
Single-shaft	1.1
Two-shaft	1.0
Three-shaft	0.9

**Table 4:** LM2500-30 compressor design parameter.

Parameter	Unit	Literature
Mass flow	Kg/s	65.8
CDP	KPa	1722
Power	KW	20134
EGT	°C	504

**Table 5:** LM2500-30 compressor operating line data (General Electric, 1981).

$N_{1}/\sqrt{\theta_{1}}$	*P*_2_/*P*_1_	$W_{1}\sqrt{\theta_{1}}/\delta_{1}$	Δ*T*_12_/*T*_1_
9450	18.06	147.5	1.530
9160	17.21	144.0	1.439
8971	16.25	137.8	1.389
8813	15.30	131.6	1.343
8660	14.37	125.5	1.307
8508	13.44	119.2	1.262
8364	12.45	112.2	1.220
8105	10.35	96.7	1.112
7772	7.88	76.6	0.983

## Nomenclature

*π*: Pressure ratio
$\dot{m}$: Mass flow rate
*θ*: Temperature ratio of inlet total temperature and standard sea-level temperature
*δ*: Pressure ratio of inlet total pressure and standard sea-level pressure
*n*: Rotational speed
*T*_sl_: Standard sea-level temperature
*P*_sl_: Standard sea-level pressure
*ψ*: Pressure coefficient
*C*_*p*_: Specific heat at constant pressure
PR: Stage pressure ratio
*γ*: Ratio of specific heat
*U*: Tangential blade speed
*U*_*m*,1_: Tangential blade speed at the midspan radius
*ω*: Angular speed
*r*_*m*,1_: Midspan radius
*ϕ*: = *C*_*a*_/*U* Flow coefficient
*C*_*a*_: Axial flow velocity
*T*_*OS*_: Total temperature at the inlet
*A*_ef_: Effective section area
*σ*: Temperature coefficient
*η*: Efficiency
*R*: Gas constant
*M*: Mach number
*N*: Compressor shaft speed
*α*: Inlet flow angle
*P*: Pressure
*C*_*ϕf*_: Ratio of fouling flow coefficient
*C*_*ψf*_: Ratio of fouling pressure coefficient
*C*_*ηf*_: Ratio of fouling efficiency coefficient
*T*: Operating time
*z*: Number of infinitesimal stage
FS: Fouling severity coefficient
*c*_*g*_: Configuration coefficient.

### Subscripts and Superscripts

∗: Stagnation index
in: Inlet
out: Outlet
ref: Reference value
*f*: Fouling
1st: The first section
2nd: The second section
*R*: Reference.
